# Effect of SNHG11/miR-7-5p/PLCB1 Axis on Acute Pancreatitis through Inhibiting p38MAPK Pathway

**DOI:** 10.3390/cells12010065

**Published:** 2022-12-24

**Authors:** Tian-Jiao Song, Jun Ke, Feng Chen, Jiu-Yun Zhang, Chun Zhang, Hong-Yi Chen

**Affiliations:** 1 Shengli Clinical Medical College, Fujian Medical University, Fuzhou 350001, China; 2 Department of Emergency, Fujian Provincial Hospital, Fuzhou 350001, China; 3 Fujian Provincial Key Laboratory of Emergency Medicine, Fujian Provincial Institute of Emergency Medicine, Fujian Emergency Medical Center, Fuzhou 350001, China; 4 Department of Hepatobiliary and Pancreatic Surgery, Mindong Hospital, Ningde, Fujian Medical University, No. 89, Heshan Road, Fuan 355000, China

**Keywords:** acute pancreatitis, LncRNA-SNHG11, miR-7-5p, PLCB1, p38MAPK

## Abstract

Acute pancreatitis (AP) is an inflammatory disease of the pancreas. A growing number of studies have shown that long noncoding RNAs (lncRNAs) play an important role in AP progression. Here, we aimed to elucidate the role of Small Nucleolar RNA Host Gene 11(SNHG11) and its underlying molecular mechanisms behind AP progression. The in vivo and in vitro AP cell models were established by retrograde injection of sodium taurocholate and caerulein stimulation into AR42J cells and HPDE6-C7 cells, respectively. A bioinformatics website predicted the relationship between SNHG11, miR-7-5p, and Phospholipase C Beta 1(PLCB1) and validated it with a dual-luciferase reporter assay and an RNA immunoprecipitation (RIP) assay. AR42J cells and HPDE6-C7 cells were transfected with an overexpression of plasmids or shRNA to investigate the effects of the SNHG11/miR-7-5p/PLCB1 axis on cell proliferation and apoptosis, inflammatory cytokine secretion, and acute pancreatitis. Low expression of SNHG11 and PLCB1 and high expression of miR-7-5p were observed in AP pancreatic tissue and AP cell models. SNHG11 overexpression inhibited apoptosis and inflammatory responses induced by caerulein. Simultaneously, we discovered that SNHG11 regulates PLCB1 expression by sponging miR-7-5p. PLCB1 overexpression abrogated inflammatory damage exacerbated by miR-7-5p enrichment. In addition, the SNHG11/miR-7-5p/PLCB1 axis could be involved in caerulein-induced inflammatory injury by participating in the p38MAPK signaling pathway. The overexpressed SNHG11/miR-7-5p/PLCB1 axis can inhibit AP progression by participating in the p38MAPK signaling pathway, thereby providing a potential therapeutic target and therapeutic direction for AP therapy.

## 1. Introduction

Acute pancreatitis (AP) is one of the most common clinical diseases [1], affecting 20 to 80 people per 100,000 every year [2,3], which varies from country to country, and the Atlanta classification divides AP into three grades based on their severity, including mild, moderate and severe [4]. Although mild cases are self-limiting and have a good prognosis, about 20% to 30% of patients can develop severe acute pancreatitis (SAP) [5,6] Exploring the mechanism by which mild pancreatitis progresses to severe pancreatitis has gradually become a hot topic [7]. SAP is a critical illness that progresses quickly, resulting in multiple organ failures in patients. Even after active treatment, the fatality rate remains as high as 30% [8,9,10]. Treating severe acute pancreatitis and its local and systemic complications is quite difficult. Due to the lack of specific therapy, many people gradually realize that studying the mechanism of mild to severe pancreatitis is of great significance. There is a significant gap in understanding how acute pancreatitis rapidly progresses to severe pancreatitis. We need to study its pathogenesis and research potential therapeutic targets.

In recent years, with the popularization of high-throughput sequencing technology, the role of noncoding RNAs in various human diseases, has been paid more attention, including long noncoding RNAs (lncRNAs) and microRNAs (miRNAs) [11,12]. More than 200 nucleotides in length characterized lncRNAs, and their roles span multiple biological processes [13]. LncRNAs are abnormally expressed in various human diseases and play an important role in stimulating pathogenesis and maintaining disease progression [14]. Therefore, it was gradually discovered that developing mRNA vaccines targeting lncRNAs is of great significance for clinical treatment. Increasing evidence also confirms that lncRNAs are key regulators of inflammatory responses and inflammation-related diseases [14,15,16] (such as sepsis [17]). For example, FOXF1-adjacent noncoding developmental regulatory RNA (FENDRR) has been investigated in recent studies for its potential regulatory role in gastrointestinal diseases such as prostate cancer [18] and gastric cancer [19]. SNHG11, a small nucleolar RNA hostgene11, is a newly discovered LncRNA located on human chromosome 20q11.23 [20,21]. SNHG11 has been linked to the occurrence and development of various tumors [22,23,24,25], but its mechanism and role in the progression of pancreatitis are still unclear. Therefore, it is important to investigate the role of the novel SNHG11 in the progression of pancreatitis and its potential therapeutic intervention.

As an interesting class of RNAs, lncRNAs have been shown to bind to various targets, thereby controlling cellular processes and downstream gene expression [26]. Competing endogenous RNA (ceRNA) is one of the important ways for lncRNA to regulate downstream gene expression by interacting with miRNA [27]. MiRNAs are indispensable regulators of biological processes and are approximately 22 nucleotides in length [28]. It is known that many microRNAs can lead to gene silencing by binding to mRNA, and the mechanism of ceRNA is to regulate gene expression by competitively binding microRNAs. Furthermore, ceRNA can bind to microRNA through response elements (microRNA response elements, MREs) to disable microRNA, which reveals a potential mechanism of lncRNA to regulate downstream gene expression, which has great biological significance [29]. In recent years, an increasing number of studies have confirmed the importance of miRNAs, such as miR-21-3p and miR-155, in the development of AP [30,31]. miRNA-7-5p is a microRNA discovered in recent years, and studies have shown that miR-7-5p plays an important role in intestinal epithelial barrier damage and repair [32]. Another study reported that curcumin could prevent brain damage and cognitive dysfunction during ischemia-reperfusion by regulating the expression of miR-7-5p [33]. However, the reports of the relationship between miR-7-5p and the progression of pancreatitis are still few, therefore it is of practical significance to explore the role and mechanism of miR-7-5p in the progression of pancreatitis.

Changes in many important pathways accompany the progression of pancreatitis. Among them, the p38MAPK signaling pathway has been confirmed to play an important role in acute pancreatitis [34]. Mitogen-activated protein kinases (MAPKs) are a class of serine/threonine protein kinases and signal transduction mediators [35]. Three major MAPK families are found in mammalian cells, including p38MAPK, extracellular signal-regulated kinases (ERK1/2), and c-Jun amino-terminal protein kinase (JNK) [36]. Among them, studies have shown that p38MAPK affects the severity of pancreatitis. Moreover, in the development of acute pancreatitis, the p38MAPK signaling pathway regulates NF-κB activation and plays a crucial role in the inflammatory cascade [37]. In addition, the activation of p38MAPK leads to the phosphorylation of the downstream protein kinase MK2 of p38MAPK, which triggers the expression of NF-κB and increases the inflammatory response [38]. Furthermore, drugs such as Emodin and Salvia miltiorrhiza have been found to exert their effects through the p38MAPK signaling pathway in treating pancreatitis. Therefore, searching for the related regulatory mechanism of the p38MAPK signaling pathway in pancreatitis and the development of potential combination therapy have practical significance for treating pancreatitis in the future.

In conclusion, this article shows the role of the lncrna-SNHG11/mir-7-5p/PLCB1 axis in pancreatitis for the first time, and once again, confirms the important role of non-coding RNA in pancreatitis. Our results demonstrate the potential mechanism for the progression of mild pancreatitis to severe pancreatitis and provide a new theoretical basis for new clinical treatment options and early intervention for acute pancreatitis.

## 2. Materials and Methods

### 2.1. Establishment of a Rat Model of AP

Male Sprague-Dawley rats weighing 225 ± 250 g were purchased from the Experimental Animal Center of the Chinese Academy of Sciences (Shanghai, China). AP model was developed by retrograde injection of sodium taurocholate in rats. AP-modeled rats were briefly treated with a retrograde injection of sodium taurocholate and fasted for 12 h before the experiments with free access to water. Rats were injected with 40 mg/kg sodium pentobarbital (Beijing Biolab Technology Co., Ltd., Beijing, China), fixed on the table, routinely shaved, disinfected, and draped. Laparotomy was performed through a midline incision. After exposing the duodenum and pancreatic head, a hole was drilled in the mesenteric vessel with a No. 5 needle. A catheter was inserted through the hole in the duodenum and moved retrograde towards the pancreatic head. Microvascular clamps were applied to temporarily clamp the upper and lower ends of the biliary and pancreatic ducts. After connecting the tube end with the transfusion converter, 3% sodium taurocholate (0.1 mL/100 g; 115903; Shanghai Chemical Science and Technology Co., Ltd., Shanghai, China) was injected into rats. After 4–5 min of injection, the microvascular clamp and the catheter were removed, and the hole in the lateral duodenum wall was sutured. The equipment was removed, and a laparotomy was performed. Rats in the sham group were injected with the same amount of normal saline in the same way.

### 2.2. Establishment of AP In Vitro Cell Model in AR42J and HPDE6-C7 Cells

AR42J and HPDE6-C7 cell lines were induced by caerulein to establish an in vitro model of AP. The cells were stored in Ham’s F12 medium (Invitrogen; Thermo Fisher Science, Austin, TX, USA) supplemented with 10% fetal bovine serum (FBS, Hangzhou Sijiqing Company, Zhejiang, China), penicillin 100 U/mL, and streptomycin 100 μ/mL. Cells were seeded at 1 × 105 cells/mL density in a 6-well culture dish and incubated in a humidified incubator at 37 °C with 95% air and 5% CO_2_. The cells were treated with 100 NM caerulein dissolved in PBS, and cells treated with PBS alone were used as control. The cells were incubated in the incubator for 24 h. For a subsequent experiment, cells and culture supernatant was collected. All groups were given three triplicates.

### 2.3. HE Staining

Pancreas tissues were fixed in 4% paraformaldehyde for 24 h. Fixed tissues were dehydrated using gradient ethanol, embedded with paraffin, and sliced with 4-μm thickness. The sections were dewaxed in xylene, rehydrated in upgraded ethanol, washed three times with distilled water, and immersed in hematoxylin for 5 min. Next, sections were washed two times, differentiated using 1% HCl, and stained with eosin for 4 min. After staining, sections were dehydrated and differentiated in ethanol, and tissue morphological changes were observed using microscopy. Each experiment was repeated three times. Pathological alterations of tissues were scored, according to the edema, infiltration of inflammatory cells, hemorrhage, and pancreatic necrosis of pancreatic tissues, with 0 as no alteration, 1 as mild, 2 as moderate, 3 as severe, and 4 as very severe.

### 2.4. Enzyme-Linked Immunosorbent Assay (ELISA)

Levels of serum amylase (AMY) and lipase (LIPA) were determined using an automatic biochemical analyzer and ADVIA 2400 clinical chemistry system (Siemens AG, Berlin, Germany) according to the manufacturer’s protocols of specific commercial ELISA kits, tumor necrosis factor (TNF)-α (Ebioscience Inc., San Diego, CA, USA catalog: #BMS622), Interleukin (IL)-1β (Ebioscience Inc. San Diego, CA, USA. catalog: #BMS630) and IL-6 (Ebioscience Inc. catalog: #BMS625) levels were quantified.

### 2.5. Trypan Blue Exclusion Method

Trypan blue exclusion method is one of the earliest and simplest viability assays. Trypan blue is a negatively charged dye that only stains cells with a compromised cell membrane, indicating cell death. In contrast, viable cells lack trypan blue due to the cell membrane and dye being negatively charged.

### 2.6. Lentivirus Vector Construction and Infection

Plasmids for lentivirus packaging were purchased from Shanghai Genechem Inc. (Shanghai, China). Plasmids were transfected in HEK-293T cells using Lipofectamine 3.0 (Invitrogen, CA, USA) following the manufacturer’s instructions. The lentivirus supernatant was collected 48 and 72 h after transfection, and cells were transfected with lentivirus. The AR42J and HPDE6-C7 cell lines with stable gene expression were selected in a culture medium supplemented with puromycin (1 μg/mL; Sigma-Aldrich, St. Louis, MO, USA).

### 2.7. Cell Counting Kit-8 (CCK-8) Assay

AP cells in the logarithmic growth phase were counted, and 200 µL cells were seeded in 96-well plates at 3 × 10^3^ cells/well density. After the cells were attached to the wall, the CCK-8 reaction solution (341-07761) was added into wells at 24 h, 48 h, and 72 h, respectively, and then incubated in an incubator for 2 h. Each well’s absorbance value (OD) was detected at 450 nm of the marker. Seven parallel wells were set in each group, and the experiment was repeated three times.

### 2.8. RNA Extraction and Quantitative Real-Time PCR Analysis

For RNA extraction, total RNA was isolated using Trizol reagent (Invitrogen, Grand Island, NY, USA). In total, 1 µg of RNA was subjected to reverse transcription using Superscript III transcriptase (Invitrogen, CA, USA). Quantitative real-time PCR (qRT-PCR) was conducted using a Bio-Rad CFX96 system with SYBR green to determine the mRNA expression level of a gene of interest. The expression levels were normalized to GAPDH RNA expression. Quantitative real-time PCR (qRT-PCR) was conducted using a Bio-Rad CFX96 system with SYBR green to determine the mRNA/lncRNA/miRNA expression level of the gene of interest.

### 2.9. RNA Sequencing and Public Datasets

Three pairs of acute pancreatitis tissues and normal tissues were selected for RNA-sequencing analysis at Wuhan Contest Biotechnology Co., Ltd. (Wuhan, China). The public datasets used in this study included three GEO datasets (http://www.ncbi.nlm.nih.gov/geo/, GSE120138 accessed on 6 February 2022).

### 2.10. Western Blot Analysis

In brief, proteins were isolated from cells and tissues using RIPA buffer (Solarbio, Beijing, China) supplemented with proteinase inhibitors, and the protein concentration was determined with a BCA reagent (Beyotime, Beijing, China). Cell lysates were separated on SDS-polyacrylamide gels and then transferred onto polyvinylidene difluoride (PVDF) membranes (Millipore, MA, USA). After the membranes were blocked in 5% skim powdered milk for 2 h, they were incubated with primary antibodies overnight at 4 °C. The primary antibodies used in this study included: p38MAPK (Cat. No. ab170099, Abcam, Cambridge, UK), p-p38MAPK (Cat. No. ab195049, Abcam, Cambridge, UK), GAPDH (Cat. No. ET1601–4, HUA BIO, Hangzhou, China). Next, the membranes were incubated with secondary antibodies (HUABIO, Hangzhou, China) at room temperature for 1 h. The targeted proteins were visualized using enhanced chemiluminescence (ECL) reagent after three washes (Millipore, MA, USA). GAPDH was used as the loading control in this study.

### 2.11. RNA Immunoprecipitation (RIP)

RNA immunoprecipitation (RIP) assay was performed using Magna RIP™ RNA-binding protein immunoprecipitation kit (Millipore, Billerica, MA, USA) following the manufacturer’s protocol. First, transfected cells were lysed in complete RNA immunoprecipitation lysis buffer after being transfected with miR-7-5p mimics or negative control. Then, the cell extract was incubated with magnetic beads conjugated with anti-Argonaute 2 (AGO2) or anti-IgG antibody (Millipore, Billerica, MA, USA) for 6 h at 4 °C. Next, the beads were washed and incubated with Proteinase K to remove proteins. Finally, isolated RNA was extracted using TRIzol Reagent (Thermo Fisher Scientific, Waltham, MA, USA), then the purified RNA was subjected to agarose gel electrophoresis and qRT-PCR analysis.

### 2.12. Bioinformatics Analysis

The public dataset GSE120138 was retrieved from the Gene Expression Omnibus (GEO, https://www.ncbi.nlm.nih.gov/geo/, accessed on 13 February 2022) database. Differential expression analysis was performed using the limma R package with Fold change >2 or <0.5 and *p*-value < 0.05 as the screening criterion. The downstream miRNAs of lncRNA-SNHG11 were predicted through ENCORI (ClipExpNum ≥ 1, https://starbase.sysu.edu.cn/, accessed on 13 February 2022), ANNOLNC (http://annolnc.gao-lab.org/, accessed on 23 June 2022), Diana (https://diana.e-ce.uth.gr/lncbasev3, accessed on 16 February 2022) and then intersected miRNAs served as a candidate miRNA. The downstream target genes of miR-7-5p were predicted through ENCORI (ClipExpNum ≥ 1, https://starbase.sysu.edu.cn/, accessed on 16 February 2022), Targetminer (https://www.isical.ac.in/~bioinfo_miu/targetminer20.html, accessed on 16 February 2022), and TargetScan (Conserved sites ≥ 1, http://www.targetscan.org/, accessed on 16 February 2022), and then intersected with the downstream target genes of miR-7-5p to identify the candidate gene. 

### 2.13. Statistical Analysis

The data were expressed as the mean ± SD (standard deviation). The significance of changes among groups was evaluated using ANOVA with a post-hoc Tukey’s test. A *p*-value of less than 0.05 was considered statistically significant.

## 3. Results

### 3.1. Construction of Experimental Models of Pancreatitis In Vitro and In Vivo

**Figure 1:**
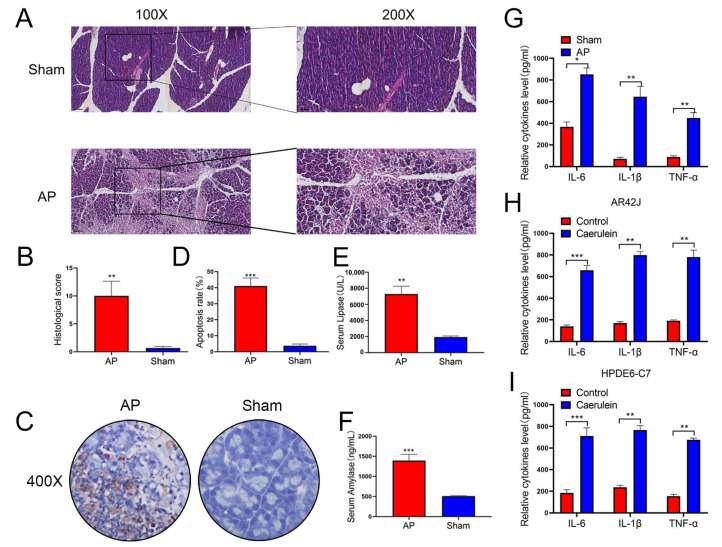
Successful establishment of a model of AP in Rat and cell lines. (**A**,**B**) The establishment of the rat model of acute pancreatitis was confirmed by HE staining, the pathological scoring was performed in two groups, *n* = 5 in each group, and representative images have been shown. (**C**,**D**) Apoptosis of pancreatic tissues in rats evaluated by TUNEL staining (*n* = 5). (**E**,**F**) Serum levels of AMY and LIPA of rats examined by ELISA (*n* = 5). (**G**–**I**) Expression of IL-6, TNF-α, and IL-1β in the rats, AR42J, and HPDE6-C7 assessed by ELISA (*n* = 3). The data are presented as means ± SDs, * *p* < 0.05; ** *p* < 0.01; *** *p* < 0.001, between the indicated groups.

The construction of AP models is already a very mature technical method [39], this study mainly used rats, AR42J, and HPDE6-C7 to construct a rat and cellular pancreatitis model. After injecting bile acids into rats, we confirmed the successful establishment of the rat pancreatitis model, the histopathological and histological changes of pancreatic tissues in rats were detected by H&E staining (Figure 1A,B). Next, we applied the TUNEL assay to study the apoptosis of pancreatic tissues in AP rats and found that apoptosis of pancreatic tissues was increased in AP rats (Figure 1C,D). In addition, Serum levels of AMY and LIPA increased in AP rats (Figure 1E,F). Then we used the extracted pancreatic inflammatory tissue and normal tissue to perform ELISA experiments to detect the inflammatory factors IL1-beta, IL6, and TNF-alfa. The results also confirmed the rat pancreatitis model of successful builds (Figure 1G). Finally, we added 10umol/l caerulein to AR42J and HPDE6-C7 cells and detected inflammatory factors IL1-beta, IL6, and TNF-alfa after 24 h of intervention confirmed the successful construction of the cell pancreatitis model (Figure 1H,I).

### 3.2. lncRNA-SNHG11 Can Delay the Progression of Pancreatitis

**Figure 2:**
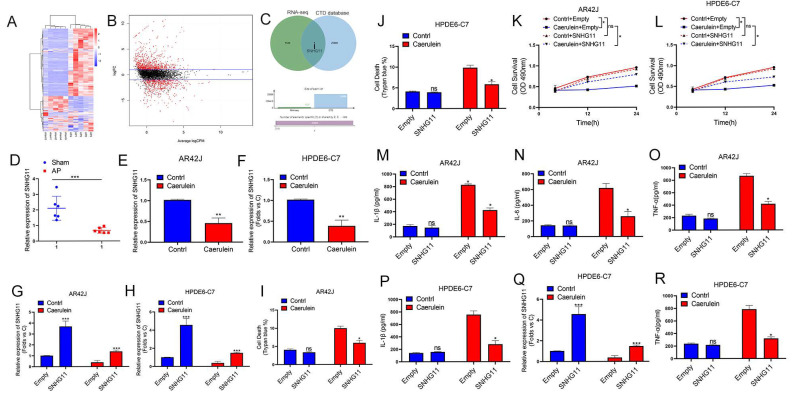
Pancreatitis is accompanied by a decrease in lncRNA-SNHG11. (**A**,**B**) The matrix (**A**) and volcano (**B**) maps of differential lncRNA obtained by RNA-seq in AP vs. sham. (**C**) The CTD database was used to screen pancreatitis-related genes and intersect with different lncRNAs to find the common gene SNHG11. (**D**–**F**) Expression of SNHG11 in the rats, AR42J and HPDE6-C7 assessed by qPCR (*n* = 3). (**G**,**H**) The stable overexpression strain of SNHG11 was constructed in AR42J and HPDE6-C7 cells, and the transfection effect was verified by qPCR experiment (*n* = 3). (**I**,**J**) The effect of SNHG11 on cell death induced by caerulein was observed by Trypan blue test in AR42J and HPDE6-C7(*n* = 3). (**K**,**L**) Cell proliferation was assessed by CCK8 assay in AR42J and HPDE6-C7 cell lines (*n* = 3). (**M**–**R**). Expression of IL-6, TNF-α, and IL-1β in the AR42J and HPDE6-C7 cell lines (empty vs. SNHG11) assessed by ELISA (*n* = 3). The data are presented as means ± SDs, * *p* < 0.05; ** *p* < 0.01; *** *p* < 0.001, between the indicated groups. ns = no significance.

Using the successfully constructed rat pancreatitis model, we investigated the role of lncRNAs in pancreatitis progression. First, we used rat pancreatitis tissues and normal tissues to find differences of lncRNAs by RNA-seq (Figure 2A,B) and used the Comparative Toxicogenomics Database (https://ctdbase.com/, accessed on 7 March 2022) to screen pancreatitis disease-related genes (Figure 2C), our results showed that lncRNA-SNGH11 might be closely related to pancreatitis disease progression. We then further verified the relationship between the expression of SNHG11 and the progression of pancreatitis. Our results demonstrated decreased SNHG11 in both rat and cellular pancreatitis models (Figure 2D–F). As a result, we created SNHG11 stable overexpression strains using AR42J and HPDE6-C7 for further functional validation, and we confirmed the transfection effect in control and pancreatitis models (Figure 2G,H). Interestingly, we found that overexpression of SNHG11 in normal cells did not significantly affect cell death and proliferation. However, overexpression of SNHG11 significantly inhibited cell damage (Figure 2I–L) caused by caerulein. Our ELISA experiments also confirmed that overexpression of SNHG11 could delay the elevation of inflammatory factors (Figure 2M–R) caused by caerulein. These results suggest that SNHG11 is a lncRNA closely related to the progression of pancreatitis, and overexpression of SNHG11 can delay the progression of pancreatitis.

### 3.3. SNHG11 Can Bind to miR-7-5p in a Pancreatitis Model

**Figure 3:**
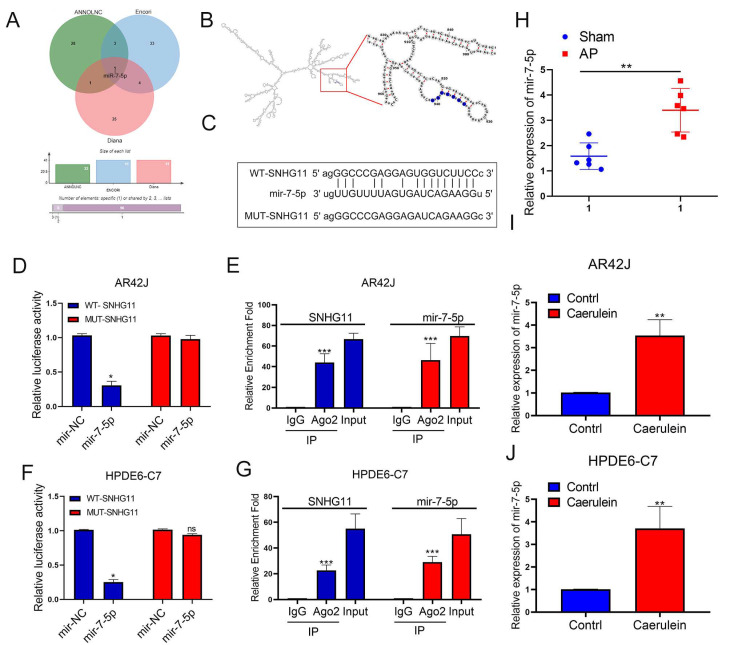
MiR-7-5p binds to lncRNA-SNHG11 in pancreatic cells. (**A**) Prediction of potentially downstream miRNAs bound to SNHG11 by Annolnc, Encori, and Diana databases. (**B**) Prediction of the binding position of SNHG11 and miR-7-5p through the secondary structure of SNHG11. (**C**) Schematic diagram of the MUT-SNHG11 plasmid. (**D**–**G**) The relationship between SNHG11 and miR-7-5p was verified by dual-luciferase reporter assay and RIP assay in AR42J and HPDE6-c7 cells. (**H**–**J**) The expression of miR-7-5p was assessed in rat tissues, AR42J, and HPDE6-C7 cells. The data are presented as means ± SDs, *n* = 3 for each group, * *p* < 0.05; ** *p* < 0.01; *** *p* < 0.001, ns = no significance between the indicated groups.

Following that, we hoped to delve deeper into the potential mechanism by which SNHG11 influences pancreatitis progression. Based on our literature reading, we hypothesized that ceRNA is an important mechanism for lncRNA to regulate downstream gene expression. Therefore, we first searched for the potential downstream miRNA targets of SNHG11. We used ANNOLNC, ENCORI, and DIANA databases to make predictions. We found that miR-7-5p may be downstream of SNHG11 (Figure 3A), and the binding position of SNHG11 and miR-7-5p was predicted through the secondary structure. (Figure 3B). Subsequently, we confirmed the binding effect of SNHG11 and miR-7-5p. To create a new mut-SNHG11, we replaced the binding sites of SNHG11 and miR-7-5p (Figure 3C), which was used in subsequent Lucifer experiments. It was confirmed that SNHG11 binds to miR-7-5p through this site (Figure 3D,F), and our RIP experiments also confirmed the binding of SNHG11 and miR-7-5p to AGO2 (Figure 3E,G), which also suggested the existence of a ceRNA mechanism between SNHG11 and miR-7-5p. We subsequently explored the expression of miR-7-5p in rat and cellular models of pancreatitis and showed that miR-7-5p was significantly upregulated in pancreatitis progression (Figure 3H–J).

### 3.4. miR-7-5p Is an Important Downstream of SNHG11 in the Progression of Pancreatitis

**Figure 4:**
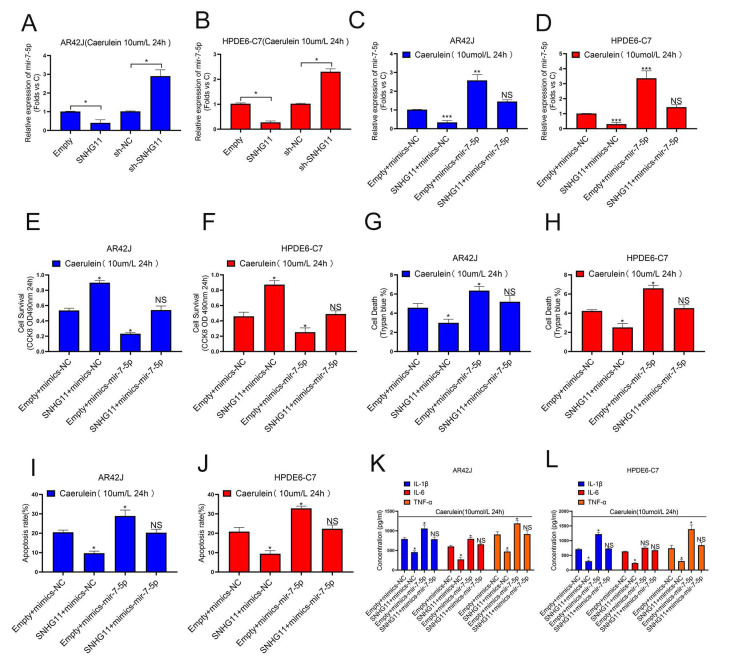
LncRNA-SNHG11 plays a role in pancreatitis through MiR-7-5p. (**A**,**B**) The regulation effect of SNHG11 expression on MiR-7-5p was observed in AR42J and HPDE6-C7 cells. (**C**,**D**) The expression of miR-7-5p in different pancreatitis cell models was verified. (**E**,**F**) Cell proliferation was assessed by CCK8 assay in AR42J and Hpde6-C7 Cell models. (**G**–**J**) The effects of SNHG11 on cell death and apoptosis in AR42J and HPDE6-C7 cell models were observed by Trypan blue assay and Flow cytometry assay. (**K**,**L**) Expression of IL-6, TNF-α, and IL-1β in the AR42J and HPDE6-C7 cell lines assessed by ELISA. The data are presented as means ± SDs, *n* = 3 for each group, * *p* < 0.05; ** *p* < 0.01; *** *p* < 0.001, NS = no significance, between the indicated groups.

Because our findings in Figure 2 suggest that SNHG11 is primarily involved in pancreatitis models, our subsequent research will focus on pancreatitis cell models. First, we explored the relationship between SNHG11 and miR-7-5p in the successfully constructed AR42J and HPDE6-C7 pancreatitis models, and the results showed that both overexpression and knockout of SNHG11 significantly changed the expression of miR-7-5p (Figure 4A,B). To further explore whether SNHG11 exerts its effect on miR-7-5p, we performed four interventions in pancreatitis cell models, namely empty+mimics-NC, SNHG11+mimics-NC, empty+mimics-miR-7-5p, SNHG11+mimics-miR-7-5p, and verified the differential change of miR-7-5p between them (Figure 4C,D). Following that, we investigated the functional changes of the cells using these four different cellular pancreatitis models. Our results show that the apoptosis-delaying effect of SNHG11 in pancreatitis cell models is restored after miR-7-5p overexpression (Figure 4E–J), and more importantly, SNHG11-induced inflammation in pancreatitis cell models factor alterations are also reverted by miR-7-5p (Figure 4K,L). These findings suggest that SNHG11 helps to delay pancreatitis by lowering miR-7-5p expression.

### 3.5. PLCB1 Is an Important Molecule in Delaying the Progression of Pancreatitis

**Figure 5:**
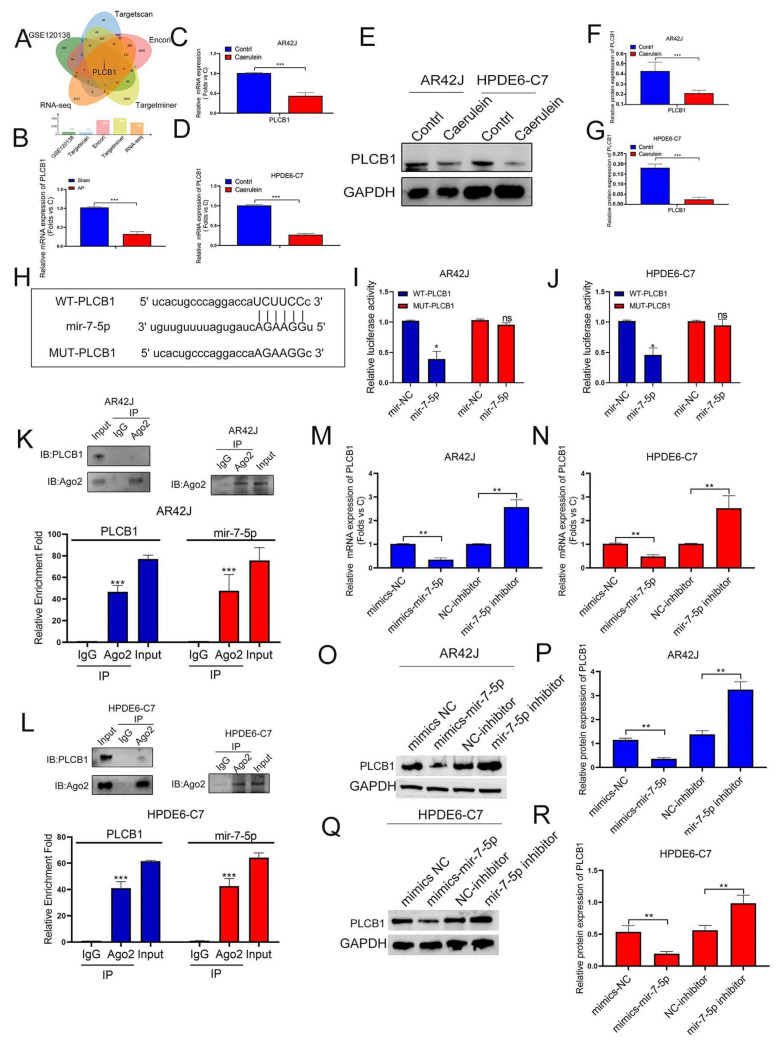
MiR-7-5p binds to PLCB-1 and regulates its expression in pancreatitis. (**A**) The potential target mRNAs of miR-7-5p were predicted in a public database and intersected with RNA-seq data to find AP-related genes. (**B**–**G**) The expression of PLCB1 was explored in rat tissues, AR42J, and HPDE6-C7 cells by Q-PCR assay and WB assay. (**H**) Schematic diagram of the PLCB1-WT and PLCB1-Mut luciferase vectors. (**I**,**J**) Relative luciferase activities in AR42J and HPDE6-C7 cells co-transfected with PLCB1-WT or PLCB1-Mut and the miR-7-5p mimic, inhibitor, or corresponding negative control. (**K**,**L**) The interaction between miR-7-5p and PLCB1 was confirmed by RIP assay in AR42J and HPDE6-C7 cells. (**M**–**R**) The regulation effect of MiR-7-5p expression on PLCB1 was observed by qPCR assay and WB assay in AR42J and HPDE6-C7 cells. The data are presented as means ± SDs, *n* = 3 for each group, * *p* < 0.05; ** *p* < 0.01; *** *p* < 0.001, ns = no significance between the indicated groups.

Combined with our previous research results, we found a ceRNA mechanism between SNHG11 and miR-7-5p, so we hope to further search for their downstream targets. We used ENCORI, TARGET SCAN, TARGETMINER, and other databases to predict the target downstream of miR-7-5p. At the same time, combined with our sequencing files (Figure 2A,B) and the microarray data of pancreatitis in GEO (GSE120138) for screening, the results suggest that PLCB1 may be a potential downstream target of miR-7-5p and be involved in the progression of pancreatitis (Figure 5A). Therefore, we then verified the role of PLCB1 in pancreatitis. The results showed that PLCB1 was significantly downregulated in pancreatitis at the transcriptional and protein expression levels (Figure 5B–G). Therefore, we further designed a mutant plasmid mut-PLCB1 (Figure 5H) of PLCB1 according to the binding sites of miR-7-5p and PLCB1 and further verified the binding of miR-7-5p to PLCB1. We first confirmed the binding sites of miR-7-5p and PLCB1 by Lucifer experiments (Figure 5I,J) and by RIP experiments to demonstrate the binding of miR-7-5p and PLCB1 (Figure 5K,L). Finally, in a cellular model, we confirmed the regulatory role of miR-7-5p on PLCB1 at the transcriptional and translational levels (Figure 5M–R).

### 3.6. PLCB1 Is a Downstream Molecule of miR-7-5p Affecting Pancreatitis Progression

**Figure 6:**
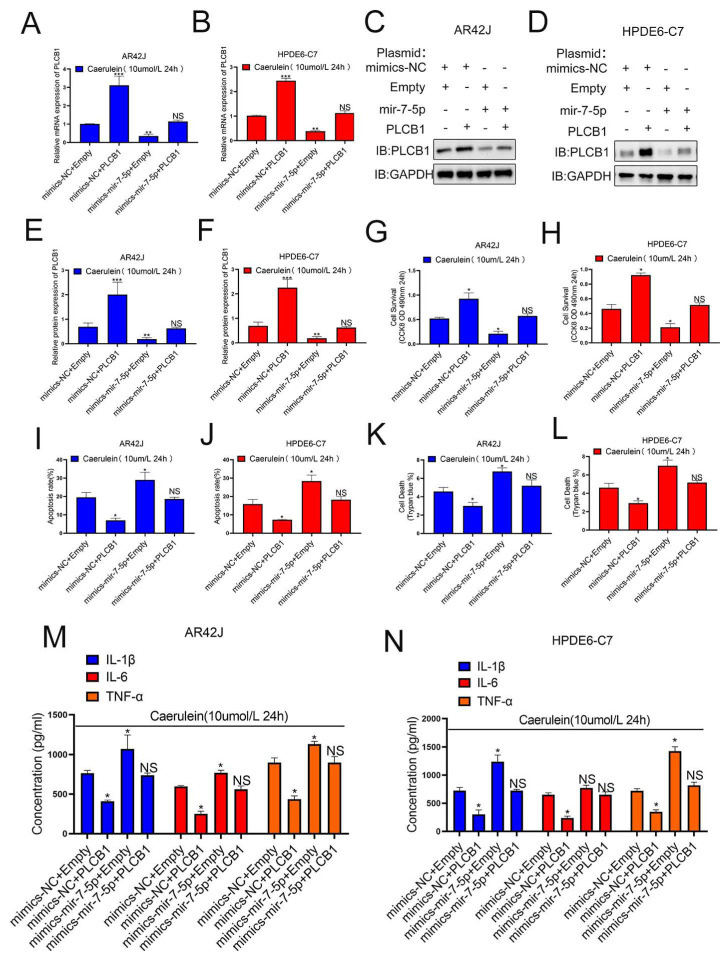
MiR-7-5p plays a promoting role through PLCB-1 in pancreatitis. (**A**–**F**) The expression of PLCB1 was verified in different cell models. (**G**,**H**) Cell proliferation was assessed by CCK8 assay in Figure 6A–F cell models. (**I**,**J**) Cell apoptosis was monitored by flow cytometry assay in Figure 6A–F cell models. (**K**,**L**) The effects of SNHG11 on cell death caused in Figure 6A–F cell models were observed by Trypan blue assay. (**M**,**N**) Expression of IL-6, TNF-α, and IL-1β in Figure 6A–F cell models were assessed by ELISA. The data are presented as means ± SDs, *n* = 3 for each group, * *p* < 0.05; ** *p* < 0.01; *** *p* < 0.001, NS = no significance between the indicated groups.

To verify whether PLCB1 is a downstream molecule of miR-7-5p affecting pancreatitis progression, we designed four subgroups (mimics-NC+empty, mimics-NC+PLCB1, mimics-miR-7-5p+empty, mimics-miR-7-5p+PLCB1), in AR42J and HPDE6-C7 cell models of pancreatitis and verified the expression of PLCB1 at the transcriptional and translational levels in the four groups (Figure 6A–F). In addition, we investigated the functional changes of the cells using these four sets of cellular pancreatitis models. Our results showed that the effect of miR-7-5p on promoting pancreatitis progression was restored by PLCB1 expression (Figure 6G–L), and the effect of miR-7-5p on promoting inflammatory factors was also restored by PLCB1 (Figure 6M,N). These results suggest that PLCB1 is a critical downstream of miR-7-5p in promoting pancreatitis progression. Therefore, our results demonstrate the role of the SNHG11/miR-7-5p/PLCB1 axis in pancreatitis progression.

### 3.7. The SNHG11/miR-7-5p/PLCB1 Axis Delays the Progression of Pancreatitis by Regulating the p38MAPK Pathway

**Figure 7:**
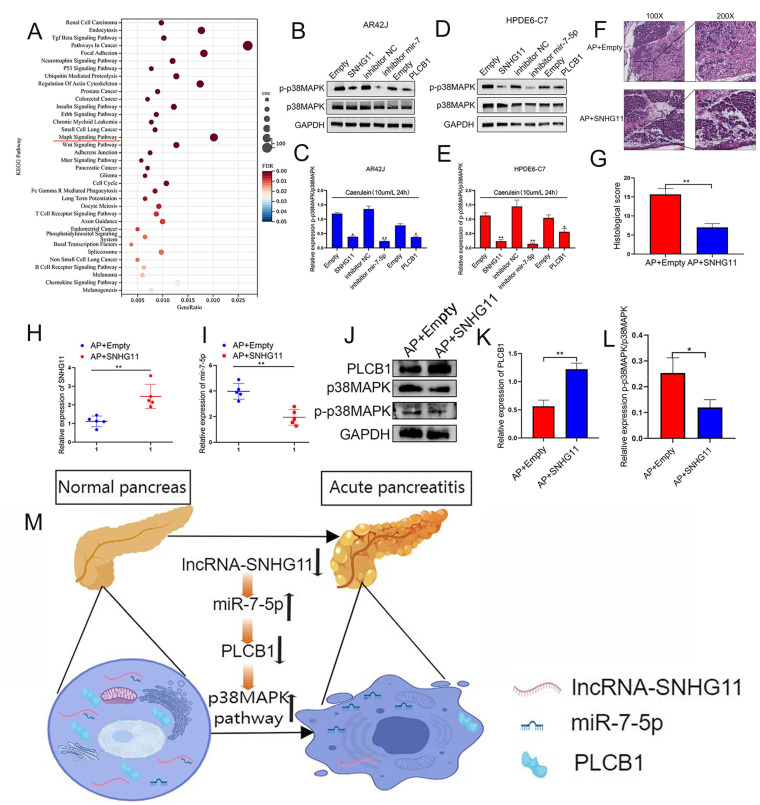
SNHG11/miR-7-5p/PLCB1 plays an inhibitory role in pancreatitis by inhibiting the p38MAPK signaling pathway. (**A**) The bubble chart shows a significant enrichment of the KEGG pathway by PLCB1-related ceRNA genes. (**B**–**E**) In AR42J and HPDE6-C7 cells, the high expression of SNHG11 and PLCB1 could inhibit the expression of p-P38MAPK and the activation of the MAPK signaling pathway, while the low expression of MiR-7-5p showed the same effect. (**F**,**G**) The effect of SNHG11 on rat models of AP was confirmed by HE staining, and representative images have been shown. (**H**,**I**) The expression of SNHG11 and miR-7-5p was assessed in different rat tissues(AP+empty vs. AP+SNHG11). (**J**–**L**) The expression of PLCB1 and *p*-P38MAPK was verified in different rat tissues (AP+empty vs. AP+SNHG11). The data are presented as means ± SDs, *n* = 3 each group, * *p* < 0.05; ** *p*< 0.01; between the indicated groups. (**M**) Schematic diagram of SNHG11/MiR-7-5p/PLCB1 affecting pancreatitis through activation of p38MAPK pathway.

We hope to investigate the specific pathways of the SNHG11/miR-7-5p/PLCB1 axis after confirming its role in pancreatitis progression. While using the ENCORI database for pathway prediction, we found that the PLCB1 protein is closely related to the p38MAPK pathway (Figure 7A). Therefore, we explored the regulatory relationship of the SNHG11/miR-7-5p/PLCB1 axis on p38MAPK, the main protein of the p38MAPK pathway. Our results demonstrate that p38MAPK is down-regulated when SNHG11 and PLCB1 are overexpressed, and p38MAPK is down-regulated when the miR-7-5p expression is inhibited (Figure 7B–E). Subsequently, we further explored the role of SNHG11 in the rat AP model. Our results show that when SNHG11 is overexpressed in the rat AP model (injection of SNHG11 adenovirus), the progression of pancreatitis can be weakened (Figure 7F,G). More importantly, SNHG11 also showed effects on miR-7-5p, PLCB1, and p38MAPK pathways in vivo (Figure 7H–L). Our results demonstrate the regulatory role of the SNHG11/miR-7-5p/PLCB1 axis on the p38MAPK pathway and preliminarily confirm the mechanism of the snhg11/mir7-5p/PLCB1 axis in delaying pancreatitis(Figure 7M).

## 4. Discussion

Acute pancreatitis (AP) is one of the most common diseases in the world. Although the treatment of mild pancreatitis has a good prognosis, once it progresses to severe pancreatitis, the disease progresses rapidly and easily, leading to multiple organ failures. Currently, the clinical treatment of pancreatitis is mainly based on anti-infection, and there is still a large gap in how acute pancreatitis progresses to severe pancreatitis. In our article, we demonstrated the role of the MIR17HG/miR-7-5p/PLCB1 axis in pancreatitis for the first time. Furthermore, we found that lncRNA-SNHG11 could attenuate the effect of miR-7-5p on the transcriptional inhibition of PLCB1 through adsorption to miR-7-5p and delay the progression of pancreatitis. Our results provide new targets and theoretical directions for treating clinical pancreatitis.

Pancreatic acinar cells are the major cell type in the pancreas and are major victims of pancreatic toxins (such as cholecystokinin [40] and bile acids [41]). Therefore, the damage to pancreatic acinar cells is also an important reason for the progression of pancreatitis. Unfortunately, no specific targets or drugs have been developed to prevent further damage to acinar cells, which may prevent the progression of acute pancreatitis to severe pancreatitis, which is an important direction. In recent years, increasing evidence suggests that lncRNAs are key regulators of inflammatory responses and inflammation-related diseases [42]. In this study, we used the constructed AP animal model to explore the role of lncRNA in the progression of pancreatitis by RNA-seq combined with bioinformatics analysis. The results showed that when pancreatitis progressed, lncRNA-SNHG11 was significantly down-regulated. Subsequently, in the in vitro experiments, the AR42J and HPDE6-C7 pancreatitis models induced by caerulein confirmed that overexpression of SNHG11 can reduce the expression of inflammatory factors IL6, IL1-β, and TNF-alfa. This is the first time SNHG11 has been shown to play a role in pancreatitis. It is also an important theoretical supplement for the role of lncRNA in pancreatitis, providing new targets and possibilities for the treatment of pancreatitis.

There are many ways for LncRNA to function, mainly: 1. Signal: to function, for example, it can act as a response to other molecular behaviors, such as transcription factors or signaling pathways, to continue to transmit signals downstream; 2. Guide: Recruit chromatin modification-related enzymes to specific target genes, cis or trans; 3. Scaffold: form nucleoproteins (ribosomes) to perform specific functions. For example, telomerase is a nuclear protein. Another example is rRNA and ribosomes [43]. The most important is that lncRNA acts as a decoy by binding to transcription factors, chromatin-binding proteins to disable it, or binding to miRNA to disable it, a “sponge” [44,45,46]. Therefore, this study explored the potential mechanism of SNHG11’s role in pancreatitis. Our results showed that SNHG11 could regulate the expression of the downstream PLCB1 gene by adsorbing MiR-7-5p. It is confirmed that SNHG11 plays a role in pancreatitis through this pathway axis (lncRNA-SNHG11/miR-7-5p/PLCB1). This is the first report of the lncRNA-SNHG11/miR-7-5p/PLCB1 pathway axis and the first report of miR-7-5p and PLCB1 genes in pancreatitis. Our findings will improve the regulatory mechanism of lncRNA and provide a new target and theoretical foundation for pancreatitis mechanism research.

Clinically, one of the important reasons for the poor clinical prognosis of severe pancreatitis is that it can rapidly cause systemic multi-organ failure. The essence of SIRS is that the body releases a variety of inflammatory mediators and cytokines to activate many physiological, biochemical, and immune pathways, causing inflammatory immune control and disorders [47]. Therefore, preventing the release of inflammatory factors IL-6, IL1-β, and TNF-alfa is an important research direction to prevent the progression of acute pancreatitis. Furthermore, when severe pancreatitis causes lung and intestinal damage, there is a significant increase in the expression of inflammatory factors on target organs. Therefore, we are committed to finding important sites that can prevent the release of inflammatory factors. In our study, we discovered the relationship between the new target molecules SNHG11 and PLCB1 genes and inflammatory cytokines in pancreatitis and confirmed the regulation of SNHG11 and PLCB1 genes on inflammatory cytokines in vitro in vivo experiments. The relationship between SNHG11 and PLCB1 genes and the progression of inflammation in vivo may be one of the important reasons pancreatitis causes systemic organ failure. More importantly, our conclusions indicate that SNHG11 and PLCB1 genes may not only be involved in acute pancreatitis and also be one of the important targets of other inflammations in the body, which deserves more exploration on the role of SNHG11 and PLCB1 genes in the disease in the future.

PLCB1 is a member of the phospholipase family of proteins, and its main function is to catalyze the generation of inositol 1,4,5-triphosphate (IP3) and diacylglycerol from phosphatidylinositol 4,5-bisphosphate [48]. PLCB1 has been linked to several diseases, including schizophrenia [49], epileptic encephalopathy [50], and myotonic dystrophy [51]. PLCB1 is also involved in the cell cycle and cell proliferation [52] and is associated with breast cancer [53] and colorectal cancer [54]. As an important member of the phospholipase family, PLCB1 plays an important role in intracellular signal transduction, and abnormal signal transduction is often one of the important reasons for disease progression and tumor progression [55,56]. However, there are few reports on PLCB1 and inflammatory progression, especially the role of PLCB1 in pancreatitis. For the first time, the role of PLCB1 in pancreatitis progression was confirmed, and it was discovered that PLCB1 could affect the release of inflammatory factors, which may be related to its important role in intracellular signal transduction. More importantly, as an important signaling pathway in the pancreatitis cascade reaction, our findings confirmed the correlation between the PLCB1 and the P38MAPK signaling pathways, opening up new avenues for future research into the PLCB1 and P38MAPK signaling pathways.

## 5. Conclusions

In conclusion, we first confirmed in vivo and in vitro that lncRNA-SNHG11 is abnormally reduced in the progression of acute pancreatitis. The reduction of this lncRNA will further reduce the adsorption of miR-7-5p, make miR-7-5p bind a large amount of downstream protein PLCB1, reduce its expression, enhance the P38MAPK signaling pathway, form a cascade effect, and promote the progression of acute pancreatitis. Our findings point to a potential mechanism for the progression of mild pancreatitis to severe pancreatitis, as well as a target and theoretical foundation for new clinical treatment options for acute pancreatitis.

## Data Availability

The original contributions presented in the study are included in the article. Further inquiries can be directed to the corresponding authors.
